# Therapeutic resistance in HPV-positive oropharyngeal squamous cell carcinoma: molecular mechanisms, clinical challenges, and precision strategies

**DOI:** 10.3389/fgene.2026.1871025

**Published:** 2026-07-29

**Authors:** Yiming Meng, Jing Sun, Yushu Ma, Cuicui Kong

**Affiliations:** 1 Department of Central Laboratory, Liaoning Cancer Hospital & Institute, Cancer Hospital of Dalian University of Technology, Shenyang, Liaoning, China; 2 Department of Biobank, Liaoning Cancer Hospital & Institute, Cancer Hospital of Dalian University of Technology, Shenyang, Liaoning, China

**Keywords:** cancer stem cells, DNA damage response, HPV-positive oropharyngeal squamous cell carcinoma, platinum resistance, therapeutic resistance

## Abstract

Human papillomavirus (HPV)-positive oropharyngeal squamous cell carcinoma (OPSCC) exhibits exceptional sensitivity to chemoradiotherapy, with 5-year overall survival exceeding 80%, thereby providing a compelling rationale for treatment de-escalation. However, 15%–20% of patients develop therapeutic resistance and locoregional recurrence, resulting in markedly inferior outcomes. This Review systematically dissects the molecular architecture of treatment resistance in HPV-positive OPSCC, with particular emphasis on mechanisms conferring resistance to radiotherapy, platinum-based chemotherapy, and EGFR-targeted therapy (cetuximab). We delineate core drivers — aberrant DNA damage response (DDR) signaling, epigenetic reprogramming, cancer stem cell (CSC) plasticity, and non-coding RNA networks—and pinpoint actionable vulnerabilities. The contributions and limitations of patient-derived organoids, genetically engineered mouse models, and pivotal clinical trials (notably RTOG 1016) are critically appraised. We address persistent controversies, including the complex relationship between de-escalation and resistance, and highlight the lack of integrative resistance biomarkers. Finally, we propose a roadmap to bridge translational gaps and accelerate the development of precision strategies that circumvent resistance.

## 1 Introduction

### 1.1 HPV-positive OPSCC as a distinct disease entity

HPV-positive OPSCC is now unequivocally recognised as a distinct clinicopathological entity within head and neck cancers, fundamentally divergent from its HPV-negative counterpart in molecular aetiology, epidemiological profile, and therapeutic responsiveness (Chen, 2025). Arising predominantly in younger, non-smoking individuals (median age 55–60 years), it is driven by persistent infection with high-risk HPV genotypes, with HPV-16 accounting for ∼90% of cases. The viral oncoproteins E6 and E7 orchestrate malignant transformation through ubiquitin-mediated degradation of p53 and retinoblastoma protein (pRb), respectively, thereby abrogating G1/S checkpoint control and precipitating unrestrained cell-cycle progression coupled with genomic instability (Rampias et al., 2009). This unique molecular aetiology confers inherently superior chemoradiosensitivity and prognosis, with 5-year overall survival exceeding 80%. However, this therapeutic advantage is not uniformly preserved across all treatment modalities or de-escalation strategies (Martinelli et al., 2025). Cetuximab was initially considered an attractive substitute for cisplatin because EGFR inhibition was expected to retain radiosensitisation while reducing treatment-related toxicity. Yet the landmark randomised trials RTOG 1016 and De-ESCALaTE showed that radiotherapy plus cetuximab was inferior to cisplatin-based chemoradiotherapy, with poorer tumour control and no meaningful reduction in severe toxicity (Gillison et al., 2019; Mehanna et al., 2019). These findings underscore that treatment sensitivity in HPV-positive OPSCC is highly pathway- and modality-dependent: whereas radiotherapy and platinum agents exploit HPV-induced defects in DNA damage response and cell-cycle control, EGFR-targeted therapy may permit the persistence or selective enrichment of resistant stem-like subclones sustained by bypass signalling and cellular plasticity (Vahl et al., 2026).

### 1.2 The chemoradiosensitivity paradox and de-escalation controversies

The intrinsic hypersensitivity of HPV-positive OPSCC to DNA-damaging modalities—both chemotherapy and radiotherapy—is mechanistically rooted in E6/E7-mediated functional inactivation of the p53 and Rb tumor suppressor pathways (Mittal and Banks, 2017). This dual checkpoint abrogation renders tumor cells exquisitely vulnerable to genotoxic stress, as they cannot mount p53-dependent G1 arrest or apoptosis and must therefore rely primarily on attenuated S/G2 checkpoints for genomic surveillance (Liu et al., 2018). Paradoxically, seminal phase III evidence from NRG/RTOG 1016 (n = 849) and De-ESCALaTE HPV (n = 334) — which compared radiotherapy plus cetuximab versus cisplatin—failed to demonstrate non-inferiority of the de-escalation strategy (Mehanna et al., 2016; Weidhaas et al., 2026). This clinical failure indicates that the radiosensitivity advantage is not universal; a subset of tumors harbors intrinsic or acquired mechanisms to evade therapeutic pressure, providing a strong impetus to dissect the molecular underpinnings of resistance.

### 1.3 Therapeutic resistance: An inadequately resolved challenge

**FIGURE 1:**
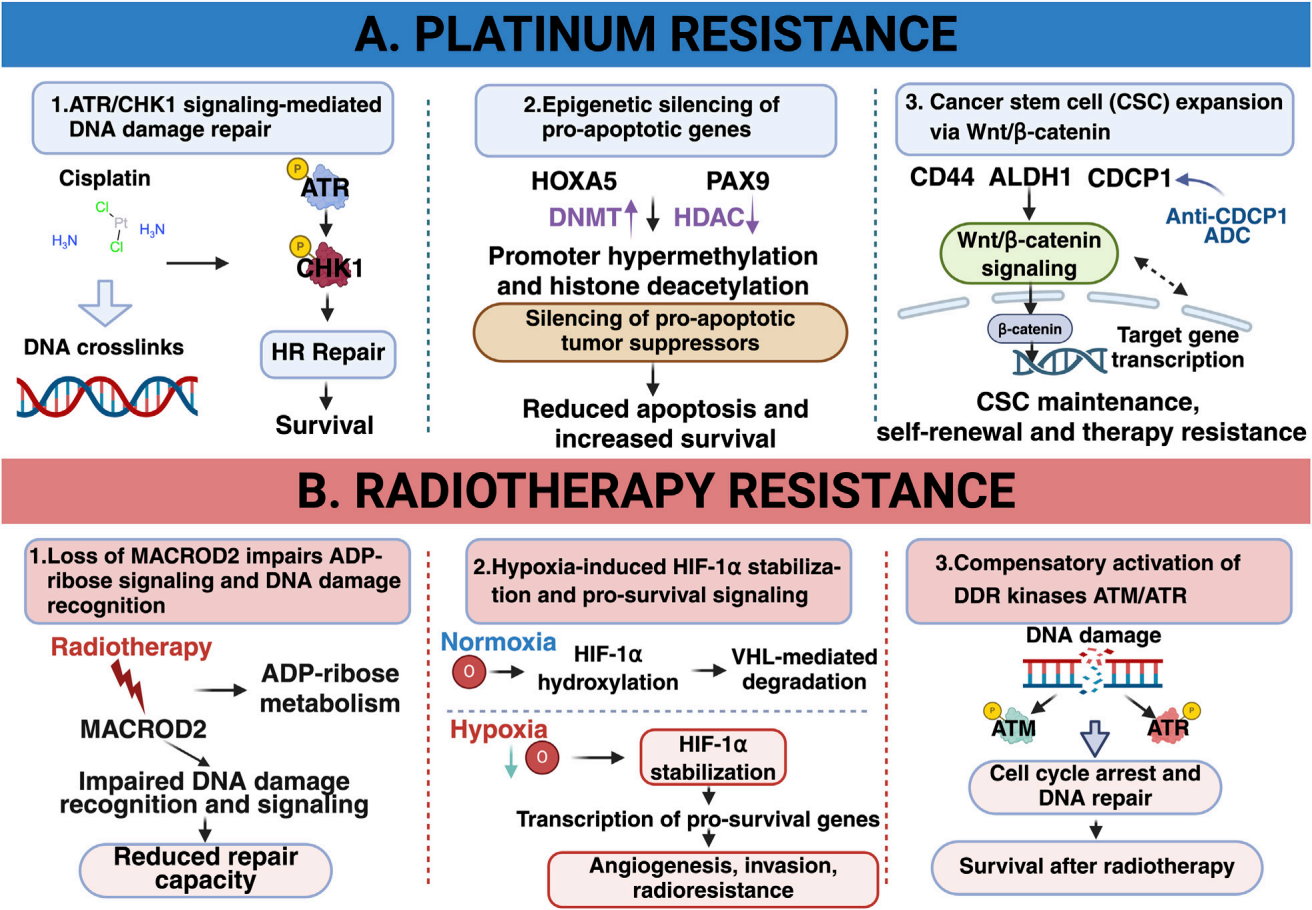
Proposed molecular mechanisms underlying therapeutic resistance in HPV-positive OPSCC. **(A)** Platinum resistance is associated with three convergent processes: activation of the ATR/CHK1 signaling axis, which enhances checkpoint control and repair/tolerance of platinum-induced DNA lesions; epigenetic silencing mediated by promoter hypermethylation of genes such as HOXA5 and PAX9; and enrichment of cancer stem cell populations marked by CD44, ALDH1, and CDCP1, supported by Wnt/β-catenin signaling. **(B)** Radiotherapy resistance involves MACROD2 deficiency with impaired ADP-ribose metabolism, stabilization of HIF-1α under hypoxic conditions with induction of downstream targets including VEGF and CAIX, and activation of ATM/ATR-centered DNA damage response signaling to facilitate checkpoint activation and DNA repair. Abbreviations: ADC, antibody-drug conjugate; ALDH1, aldehyde dehydrogenase 1; ATM, ataxia-telangiectasia mutated; ATR, ataxia-telangiectasia and Rad3-related; CAIX, carbonic anhydrase IX; CDCP1, CUB domain-containing protein 1; CHK1, checkpoint kinase 1; DNMT, DNA methyltransferase; HIF-1α, hypoxia-inducible factor 1α; MAPK, mitogen-activated protein kinase; OPSCC, oropharyngeal squamous cell carcinoma.

Although initial response rates to concurrent chemoradiotherapy are robust, 15%–20% of patients experience locoregional recurrence or distant metastasis within 5 years, and salvage outcomes remain poor (median survival <12 months). Resistance in HPV-positive disease is likely mechanistically distinct from that in HPV-negative tumors. E6/E7-driven p53 inactivation, while enhancing initial sensitivity, may force tumor cells to rely on alternative DNA repair pathways—such as ATR/ATM signaling—to maintain genomic integrity, thereby fostering resistance under selective pressure (Shamseddine et al., 2021; Zhang et al., 2025). Exemplifying this specificity, the circular RNA circ_0002722 promotes platinum resistance in OSCC through the miR-1305/YAP axis, and YAP inhibition with verteporfin restores chemosensitivity (Tsai et al., 2024), whereas CRISPR-validated loss of the mono-ADP-ribosylhydrolase MACROD2 selectively drives radioresistance without affecting cisplatin cytotoxicity (Wu et al., 2024). Although the KRAS-variant showed predictive potential for cetuximab benefit in RTOG 0522, this was not validated in RTOG 1016, underscoring the protracted path to biomarker qualification and the need for prospective cohorts (Weidhaas et al., 2026). Future studies must integrate multi-omics data, optimize preclinical models, and execute prospective trials to systematically decode resistance mechanisms (Figure 1).

### 1.4 Scope and organization of this review

This Review prioritizes mechanisms that exhibit specificity for HPV-positive OPSCC or have undergone preliminary preclinical validation, rather than exhaustively cataloguing all implicated molecular events. The scope covers three principal dimensions: (1) key molecular pathways underlying chemotherapy resistance (platinum agents and cetuximab) and radiation tolerance, including ATR/ATM signaling, YAP/TAZ, Wnt/β-catenin-driven stemness, and MACROD2 deficiency; (2) contributions of the tumor microenvironment (TME); and (3) translational lessons from clinical trials and preclinical models (Dawson et al., 2024; Shi et al., 2025; Zhang et al., 2025).

## 2 Molecular pathological hallmarks and therapeutic foundations

### 2.1 Subversion of the host cell cycle by E6 and E7

The E7 oncoprotein binds hypophosphorylated pRb with high affinity (Kd ∼4.5 nM). Through association with the cullin 2 (CUL2)-RING E3 ubiquitin ligase complex via the substrate adaptor ZER1, E7 promotes K48-linked polyubiquitination and proteasomal degradation of pRb (Jansma et al., 2014). Concomitantly, E6 forms a trimeric complex with E6-AP (UBE3A) and p53, redirecting E6-AP activity to ubiquitinate p53 at multiple lysine residues, reducing its half-life to <20 min and abolishing p53-dependent transcription of CDKN1A/p21, BAX, PUMA, and NOXA. E7 can further subvert cell-cycle control by inactivating p21 through pRb-dependent mechanisms, and by binding to and functionally inactivating p27 (Sailer et al., 2018).

### 2.2 Distinctive tumor microenvironment and immune infiltration

HPV-positive tumors exhibit a quantitatively and qualitatively distinct immune microenvironment, characterized by dense infiltration of CD8^+^ cytotoxic T lymphocytes (CTLs)—with median densities higher than in HPV-negative tumours—and elevated expression of T-cell activation markers including granzyme B, perforin, and interferon-γ (IFN-γ) (Tosi et al., 2022). This “inflamed” phenotype is mechanistically driven by persistent expression of the immunogenic viral oncoproteins E6 and E7, which serve as tumor-specific neoantigens recognized by HLA class I-restricted CD8^+^ T cells (Li et al., 2026). Notably, HPV-positive OPSCCs exhibit significantly more intense infiltration of both total (CD68^+^) and M2-like (CD163^+^) tumor-associated macrophages (TAMs) in the tumor stroma compared to their HPV-negative counterparts (Snietura et al., 2020). This M1 skewing may contribute to the superior treatment responsiveness of HPV-positive disease, although TAM plasticity under therapeutic pressure remains incompletely understood (Tong et al., 2020). Emerging evidence suggests that cancer-associated fibroblasts (CAFs) in HPV-positive tumors exhibit distinct functional profiles compared to their HPV-negative counterparts, including significantly lower αSMA^+^ CAF infiltration; however, the precise role of CAFs in modulating therapeutic responses warrants further investigation (Wang et al., 2022).

### 2.3 Molecular basis of intrinsic sensitivity: p53 and Rb pathway status

Owing to constitutive E6-mediated degradation of p53, HPV-positive tumor cells are functionally incapable of mounting p53-dependent G1/S checkpoint arrest or intrinsic apoptosis in response to genotoxic stress (Münick et al., 2025). This obligates critically dependent on the S-phase checkpoint (ATR/CHK1 axis) and G2/M checkpoint (WEE1/CDC25 axis) for genomic surveillance and DNA damage repair (Saldivar et al., 2018). Consequently, HPV-positive cells exhibit a characteristic “checkpoint addiction” phenotype, wherein pharmacological inhibition of ATR or WEE1 precipitates mitotic catastrophe due to premature mitotic entry with unrepaired DNA lesions (Diab et al., 2020). This synthetic lethal vulnerability has been validated in multiple HPV-positive HNSCC models and supports p53/Rb pathway activity as a candidate predictive biomarker, though prospective validation remains essential.

## 3 Key molecular mechanisms of chemotherapy resistance

### 3.1 Core pathways in platinum resistance

A subset of HPV-positive tumor cells adaptively upregulate the ATR/CHK1 axis under platinum pressure. ATR-mediated phosphorylation of CHK1 stabilizes stalled replication forks and facilitates homologous recombination repair of cisplatin-induced interstrand crosslinks via RAD51 and BRCA1/2 recruitment (Chen et al., 2023). Preclinical studies demonstrate that ATR pathway effector proteins—including Chk1, BRCA1, Rad51, and FANCD2—are significantly elevated in HPV-positive compared to HPV-negative HNSCC, suggesting this dependency may be amplified in HPV-positive tumors due to their reliance on S/G2 checkpoints (Guo et al., 2025). In parallel, aberrant overexpression of DNA methyltransferases (DNMT1, DNMT3A, DNMT3B) and histone deacetylases (HDAC1–3) silences tumor suppressors—such as HOXA5 and PAX9—through promoter hypermethylation, attenuating apoptosis. DNMT inhibitors (e.g., decitabine) or HDAC inhibitors (e.g., vorinostat) can reactivate these silenced loci and partially restore chemosensitivity in OSCC/HNSCC preclinical models (Mensah et al., 2021).

### 3.2 Cancer stem cells in chemoresistance

Expression of the CSC marker CD44 correlates with adverse prognosis in HPV-positive OPSCC: lack of CD44 overexpression independently predicts excellent disease-free survival in HPV16-positive patients treated with cisplatin-based chemoradiotherapy (Biesaga et al., 2021). ALDH1 has also been linked to CSC phenotypes in HNSCC, though its expression may overlap with healthy mucosal tissues, warranting cautious interpretation (Lavudi et al., 2024). Using patient-derived organoids (PDOs), Wang et al. (2026) identified CUB domain-containing protein 1 (CDCP1) as highly expressed in chemoresistant OSCC cells, where it sustains stemness through Wnt/β-catenin signaling. Notably, nanoparticle-mediated delivery of siCDCP1 effectively silenced CDCP1 expression, ablated the CSC population, and restored cisplatin chemosensitivity in resistant patient-derived xenograft (PDX) models (Wang et al., 2026). CDCP1 overexpression has been associated with poor prognosis in head and neck squamous cell carcinoma, and its role in promoting malignancy via the Wnt/β-catenin pathway has been demonstrated in nasopharyngeal carcinoma models. Additionally, transcriptomic profiling of docetaxel-resistant OSCC cell lines has identified dual specificity phosphatase 2 (DUSP2) and cytochrome P450 family one subfamily B member 1 (CYP1B1) as hub genes linked to CSC regulation and chemoresistance. Notably, DUSP2 has also been reported to be transcriptionally silenced by methylation in HNSCC, where its loss correlates with poor outcome following platinum-based chemoradiotherapy, suggesting context-dependent regulation in different resistance settings (Bie et al., 2025). Notably, DUSP2 and CYP1B1 were validated by qPCR in docetaxel-resistant OSCC cell lines, confirming their role in CSC regulation and chemoresistance. In parallel, DUSP2 is frequently silenced by promoter hypermethylation in locally advanced HNSCC, where DUSP2 methylation status, particularly when integrated with EGFR and TP53 status, serves as a candidate prognostic biomarker of clinical outcome following platinum-based chemoradiotherapy. This suggests context-dependent regulation of DUSP2 in different resistance settings (Ayyachamy et al., 2025).

### 3.3 Cetuximab resistance: The KRAS-variant and clinical correlates

Cetuximab-based de-escalation strategies proved unsuccessful in two landmark phase III trials. In RTOG 1016, radiotherapy plus cetuximab failed to demonstrate non-inferiority to cisplatin-based chemoradiotherapy for HPV-positive OPSCC(Ayyachamy et al., 2025). Similarly, the De-ESCALaTE trial showed that cetuximab offered no benefit in reducing toxicity while significantly worsening tumor control and overall survival (Mehanna et al., 2019). A retrospective analysis of RTOG 1016 b y Weidhaas et al. investigated whether the KRAS-variant, a germline mutation in a microRNA-binding site in KRAS, could identify patients with differential outcomes (Weidhaas et al., 2026). Among non-variant patients (84% of the cohort), cetuximab-based therapy was associated with significantly higher locoregional failure versus cisplatin. In contrast, KRAS-variant patients did not exhibit this increased risk, suggesting the variant may identify a subgroup for whom cetuximab is not inferior to cisplatin, potentially guiding biomarker-stratified de-escalation approaches. However, the clinical utility of the KRAS-variant for treatment selection in HPV-positive OPSCC requires independent prospective validation (Weidhaas et al., 2017).

## 4 Biological basis of resistance to radiotherapy

### 4.1 Intrinsic radiosensitivity heterogeneity: MACROD2 deficiency

Through whole genome sequencing of HPV-positive HNSCC tumors that recurred after concurrent cisplatin and radiation treatment, MACROD2 deletion was identified as enriched among treatment failures. Functional validation via siRNA and shRNA knockdown confirmed MACROD2 loss as a potent driver of radioresistance in HPV-positive HNSCC cell lines, without altering cisplatin sensitivity (Dawson et al., 2024). Mechanistically, MACROD2 depletion was associated with elevated hypoxia levels and an altered DNA damage response, providing a biological rationale for the observed radioresistance. In colorectal cancer, MACROD2 acts as a haploinsufficient tumor suppressor whose loss impairs PARP1 catalytic activity and promotes chromosome instability (Sakthianandeswaren et al., 2018). This positions MACROD2 deficiency as a promising source of radiosensitivity heterogeneity that may be particularly relevant in HPV-positive tumors; however, current evidence remains limited to *in vitro* systems and requires prospective correlative validation in tumor tissue using standardized detection methods.

### 4.2 DDR inhibitors as tumour-selective radiosensitizers across HPV-stratified HNSCC

ATR inhibitors potently enhance radiosensitivity in HPV-positive HNSCC models by abrogating CHK1-mediated G2/M arrest, thereby forcing mitotic entry with unresolved DNA lesions (Dok et al., 2021). Notably, although ATR protein levels are approximately two-fold lower in HPV-positive compared to HPV-negative HNSCC cells, ATR remains fully functional and is activated upon radiation; ATR knockdown further radiosensitizes HPV-positive but not HPV-negative cells, suggesting a narrower reserve capacity that may be therapeutically exploitable (Kohl et al., 2025). ATM inhibition also enhances the radiation response in HNSCC, though the underlying mechanisms differ. In HPV-positive cells—where the ATM-orchestrated DNA damage response is already partially impaired—ATM inhibition sensitizes cells to radiation to a lesser extent than in HPV-negative counterparts (Köcher et al., 2022). Rather than primarily impairing homologous recombination, ATM inhibition in combination with radiation predominantly induces a senescence phenotype characterized by enhanced β-galactosidase activity and secretion of senescence-associated cytokines; this senescence state may in turn facilitate immune-mediated clearance by NK cells (Jost et al., 2025). Early-phase clinical trials have begun to evaluate ATR inhibitor–radiation combinations in HNSCC. A 2026 phase I trial of berzosertib combined with cisplatin-based chemoradiotherapy in locally advanced HNSCC demonstrated safety but failed to improve complete response rates, underscoring the narrow therapeutic window (Bhatia et al., 2026). Ongoing trials, including a phase I study of camonsertib combined with stereotactic body radiotherapy for recurrent HNSCC, have incorporated HPV status as an exploratory predictive biomarker, and the ADePT-DDR platform trial is evaluating the ATR inhibitor ceralasertib with radiotherapy in curative-intent HNSCC [NCT07156227; ADePT]. Given the contrasting observations—ATR being fully functional yet its knockdown selectively radiosensitizing HPV-positive cells—randomized phase II trials in HPV-enriched cohorts are needed to define the therapeutic window and identify molecular contexts in which ATR inhibition provides meaningful clinical benefit. Beyond ATR and ATM, other DDR kinases show differential effects according to HPV status. *In vivo* shRNA screens demonstrated preferential radiosensitization by Chk1 inhibition in HPV-positive tumor models, whereas Wee1 inhibition favoured HPV-negative models (Molkentine et al., 2021). Combined inhibition of Chk1 and Wee1 radiosensitizes HPV-positive HNSCC cells more efficiently than p53-proficient normal fibroblasts, supporting a potential tumour-selective therapeutic index (Busch et al., 2017).

### 4.3 Hypoxic microenvironment and radioresistance

HIF-1α stabilization transcriptionally upregulates glucose transporters (e.g., GLUT-1) and glycolytic enzymes, shifting metabolism towards glycolysis, reducing mitochondrial reactive oxygen species, and diminishing radiation-induced apoptosis (Koukourakis et al., 2008). In HPV-positive OPSCC, this represents a departure from the typical phenotype—HPV-positive tumours generally display lower hypoxia signatures, and bioinformatic analysis has predicted that HPV16 E^7^ may inhibit HIF-1α and reduce glycolytic activity, although this finding contrasts with prior experimental evidence showing that E7 enhances HIF-1α transcriptional activity (Zhao et al., 2024). HIF-1α overexpression identifies a molecularly distinct, high-risk subgroup of HPV-positive OPSCC rather than a universal feature. Intratumoral hypoxia, reflected by HIF-1α overexpression, is associated with an increased risk of treatment failure. In HPV-positive OPSCC, HIF-1α overexpression predicts poor overall survival among patients treated with definitive (chemo)radiotherapy, independent of TNM stage. Notably, HIF-1α-positive HPV-positive patients have survival outcomes comparable to HPV-negative disease, suggesting they may not be optimal candidates for treatment de-escalation (Swartz et al., 2016). This finding has direct clinical relevance: patients with HIF-1α-overexpressing HPV-positive tumours may not be suitable candidates for treatment de-escalation. HIF-1α and its downstream targets are established predictors of radioresistance in head and neck cancer (Koukourakis et al., 2008). In HNSCC cancer stem cells, HIF-1α plays a critical role in radiation response: under hypoxic conditions, HIF-1α is expressed earlier in CSCs than in non-CSCs, suggesting that CSCs have enhanced adaptive properties to hypoxia (Wozny et al., 2017).

## 5 Influence of the tumor microenvironment and immune factors

### 5.1 Stromal remodeling and drug penetration

Activated CAFs are a major source of growth factors, collagen, and hyaluronan within the tumor microenvironment, contributing to elevated interstitial fluid pressure and the formation of a dense ECM that physically impedes drug delivery (Yang et al., 2023). Concurrently, increased matrix stiffness is sensed by integrin-mediated mechanotransduction, leading to the activation of focal adhesion kinase (FAK) and downstream pro-survival pathways, including PI3K/AKT signaling, as well as the nuclear translocation of the transcriptional co-activators YAP/TAZ, which collectively drive a drug-resistant phenotype (Wang et al., 2023; Liang et al., 2026). Crucially, the functional heterogeneity of CAFs plays a more decisive role in treatment response than total ECM abundance. For instance, myofibroblastic CAFs (myCAFs) are the subtype most closely associated with adverse prognosis in HNSCC, and a baseline ECM/myCAF gene signature was recently shown to predict non-response to PD-L1 blockade in patients with HPV-positive HNSCC. Although targeting FAK is a rational therapeutic strategy to counteract these pro-survival signals, it is important to note that clinical evidence suggests FAK inhibition with defactinib primarily reverses radio- and chemoresistance in HPV-negative HNSCC with mutant p53, rather than in p53 wild-type tumors (Pramod et al., 2026). Therefore, the efficacy of FAK inhibitors in HPV-positive OPSCC, where p53 is degraded by the viral oncoprotein E6, remains uncertain and awaits prospective validation (Pifer et al., 2024). Furthermore, the translational potential of many of these findings is currently constrained by their derivation from 2D monolayer cultures or xenograft models that fail to fully recapitulate the dynamic CAF-ECM-tumor cell crosstalk found in patients.

### 5.2 Immunoediting and immune escape

Despite abundant CD8^+^ T-cell infiltration—a hallmark of HPV-positive OPSCC—resistant tumors exhibit substantial T-cell exhaustion and immune escape through multiple complementary mechanisms (Kim et al., 2020). PD-L1 upregulation on tumor cells, together with enrichment of regulatory T cells (Tregs) and myeloid-derived suppressor cells (MDSCs), creates a profoundly immunosuppressive milieu; Tregs induce CD8^+^ T-cell exhaustion and apoptosis through PD-L1-PD-1 interaction, while M2-polarized macrophages secrete IL-6 and IL-10 that stimulate MDSC expansion, and TGF-β promotes epithelial-to-mesenchymal transition (EMT), further reinforcing resistance (Davis et al., 2016). Resistant tumors can evade CD8^+^ T-cell recognition through multiple mechanisms converging on MHC class I antigen presentation. Although the HPV E7 oncoprotein has been shown in tissue-culture models to transcriptionally downregulate MHC-I heavy-chain genes and some antigen-loading components, this pattern is not consistently observed in primary HPV-positive human tumors, which often retain or even upregulate MHC-I pathway mRNA expression (Gameiro et al., 2017). A newly discovered complementary mechanism involves the membrane-associated ubiquitin ligase MARCHF8, which degrades MHC-I post-translationally specifically in HPV-positive HNSCC(Khalil et al., 2026). Recent paired analysis of recurrent versus non-recurrent HPV-positive OPSCCs demonstrated that recurrence-prone tumors not only downregulate antitumor immunity pathways but also upregulate DNA repair pathways—a potential link between enhanced DDR and immune evasion through limiting cytosolic DNA-induced inflammation (Sannigrahi et al., 2025).

### 5.3 Targeting the TME to restore sensitivity

Low-dose cyclophosphamide, through selective depletion of regulatory T cells (Tregs), has demonstrated immunomodulatory potential in preclinical HPV vaccine models (Peng et al., 2013). Similarly, the CXCR1/2 inhibitor SX-682 significantly reduces intratumoral MDSC accumulation and enhances the tumor infiltration and effector function of adoptively transferred NK cells in HNSCC mouse models; importantly, HNSCC patients harbor elevated levels of CXCR1/2+ CD15^+^ PMN-MDSC and CD14^+^ monocytic-MDSC in both tumor tissue and peripheral blood, with tumor-resident MDSCs exhibiting greater immunosuppressive capacity (Greene et al., 2020). PEGPH20, a pegylated recombinant human hyaluronidase, enzymatically degrades intratumoral hyaluronan, thereby lowering interstitial fluid pressure, restoring microvasculature patency, and improving drug delivery (Singha et al., 2015). In HNSCC, approximately 40% of EGFR + tumors are hyaluronan-high, and PEGPH20-mediated HA depletion enhances cetuximab-dependent ADCC activity and NK cell access to tumor cells, providing a rationale for combining HA depletion with monoclonal antibody therapy (Runge et al., 2026). Collectively, these preclinical and translational studies support the biological relevance of targeting selected immunosuppressive barriers, including Tregs, MDSCs, and hyaluronan-rich stroma, in HNSCC. However, because the available evidence is derived largely from preclinical models and combination-based settings, it remains unclear whether modulation of any single microenvironmental mechanism alone would be sufficient to achieve durable clinical control in HPV-positive OPSCC. In this context, the failure of radiotherapy plus cetuximab to demonstrate non-inferiority to cisplatin-based chemoradiotherapy in RTOG 1016 highlights the challenges of translating biologically rational targeted or de-intensification strategies into effective clinical treatment for HPV-positive OPSCC. Taken together, these data more strongly support the development of biomarker-informed rational combination strategies than reliance on any single microenvironment-directed intervention alone.

## 6 Evidence from clinical trials and real-world practice

### 6.1 Lessons from RTOG 1016 and De-ESCALaTE

The landmark NRG/RTOG 1016 exposed the inadequacy of cetuximab as a de-escalation agent for HPV-positive OPSCC. In RTOG 1016 (n = 849), radiotherapy plus cetuximab failed to meet the non-inferiority criteria versus cisplatin-based chemoradiotherapy, with significantly worse overall survival (5-year OS: 77.9% vs. 84.6%), progression-free survival, and higher locoregional failure (17.3% vs. 9.9%) (Gillison et al., 2019). Similarly, the De-ESCALaTE trial (n = 334) in low-risk HPV-positive OPSCC showed significant detriment in tumour control with cetuximab (2-year OS: 89.4% vs. 97.5%; HR = 5.0; p = 0.001; 2-year recurrence: 16.1% vs. 6.0%; HR = 3.4; p = 0.0007), with no benefit in reduced toxicity (Mehanna et al., 2019). A retrospective analysis of RTOG 1016 evaluated the KRAS-variant, a germline microRNA-binding site mutation in KRAS (prevalence ∼16%), as a potential predictive biomarker. Among non-variant patients (84% of the cohort), cetuximab was associated with significantly higher locoregional failure (HR = 1.83; 95% CI 1.19–2.82). In contrast, KRAS-variant patients (16%) did not exhibit this increased risk (HR = 0.90; 95% CI 0.34–2.41), suggesting the variant may identify a subgroup for whom cetuximab is not inferior to cisplatin. However, the KRAS-variant did not reach statistical significance as a predictive biomarker for any endpoint, and its low prevalence and the need for independent prospective validation currently limit its clinical utility (Weidhaas et al., 2026).

### 6.2 Unforeseen resistance in de-escalation

The intrinsic sensitivity of HPV-positive tumors hinges on p53 and Rb disruption, whereas cetuximab merely attenuates EGFR signaling and cannot compensate for the HPV-specific DNA repair deficiencies or cell-cycle dysregulation imposed by E6/E7 (Hussain et al., 2021; Nantajit et al., 2022). The favorable prognosis associated with cisplatin-based chemoradiotherapy in HPV-positive OPSCC may be partly attributable to the elimination of therapy-sensitive tumor cells. Conversely, CD44^+^ CSC populations, which have been implicated in cisplatin resistance, may represent a reservoir for recurrence (Biesaga et al., 2021), CDCP1+ cells sustain chemoresistance through Wnt/β-catenin signaling (Wang et al., 2026), and hypoxia-induced EMT reinforces CSC stemness and migratory capacity, collectively facilitating the expansion of pre-existing resistant subclones upon reduced selective pressure. The negative outcomes of RTOG 1016 and De-ESCALaTE demonstrate that substituting cetuximab for cisplatin results in inferior survival and tumor control, establishing that cetuximab is a less effective radiosensitizer than cisplatin in HPV-positive OPSCC and should not be used as a replacement (Mehanna et al., 2020).

### 6.3 Real-world resistance complexity

Trials enroll highly selected patients with good performance status and minimal comorbidities, whereas real-world populations are often older, have heavier smoking histories, and present with comorbidities that independently influence disease trajectory. Heavier smoking was identified as the strongest smoking metric predictive of worse overall survival and disease-free survival in HPV-positive OPSCC, with >20 pack-year smokers having approximately 2-times worse survival and a greater likelihood of recurrence compared to lighter or never smokers (Chen et al., 2021). Importantly, rather than inducing additional mutational burden or recurrent oncogenic mutations, tobacco’s primary and clinically relevant biological effect in HPV-positive OPSCC is immunosuppression of the tumor immune microenvironment, characterized by low T-cell infiltration and reduced T cell-inflamed gene expression profile scores (Wahle et al., 2022). Whether smoking directly induces epigenetic reprogramming that contributes to resistance in HPV-positive tumors remains an area requiring further investigation. Real-world profiling further reveals that resistance typically arises from a confluence of mechanisms. Chemoradiation-resistant HPV-positive OPSCCs harbor higher rates of TP53 mutation, exhibit prominent activation of DNA repair, MYC, SRC, TGF-β, and PI3K-EMT-Stem pathways, and show evidence of reduced replication stress-driven anti-tumor immunity through downregulation of cytosolic dsDNA sensing (Chen et al., 2022; Guo et al., 2024). Multi-omics integrative analyses have further identified distinct molecular subtypes of HPV-associated OPSCC characterized by differential immune infiltration, transcriptional regulatory networks, and distinct mechanisms of chemoresistance (Lu, 2025). Among emerging tools to capture this complexity, circulating tumor HPV DNA (ctHPVDNA) has emerged as the most robust liquid biopsy biomarker, with pooled sensitivity of 86% and specificity of 96% for post-treatment recurrence detection—outperforming PET-CT in specificity and providing complementary surveillance utility (Campo et al., 2024). Plasma cfHPV-DNA levels decline in most patients by 3–4 weeks post-radiotherapy initiation, with approximately 30% becoming undetectable as early as this time point (fast responders). By 3–6 months post-treatment, the vast majority of patients (>95%) have cleared cfHPV-DNA, supporting the potential for longitudinal real-time monitoring (Zupancic et al., 2025). However, liquid biopsy protocols require standardization, and the sensitivity, cost-effectiveness, and optimal sampling time points require systematic evaluation in large, prospective cohorts before routine clinical implementation (Table 1 (Owadally et al., 2015; Gillison et al., 2019; Ferris et al., 2020; Yom et al., 2021; Biesaga et al., 2022; Warlow et al., 2022; Lilja-Fischer et al., 2023; Carlander et al., 2024; Lauritzenet al., 2024; Panwar et al., 2024; Jackson et al., 2025; Maity et al., 2025; Evans et al., 2026; Weidhaas et al., 2026)).

**TABLE 1:** Key clinical trials informing therapeutic resistance in HPV-positive OPSCC.

Trial	Phase	Regimen and de-escalation strategy	Key finding (efficacy and resistance)	Biomarker/Subgroup relevance	Ref.
NRG/RTOG 1016 Trial	III	Regimen: IMRT + cetuximab vs. IMRT + cisplatin; De-escalation Strategy: Attempted to reduce late toxicity in HPV + OPSCC, but trial negative	Efficacy: KRAS-variant not associated with outcomes; higher LRF in nonvariant patients with cetuximab; no OS/PFS interaction Resistance: Nonvariant patients may have lower cetuximab sensitivity	Biomarker: KRAS-variant (16% prevalence); lower acute mucositis in variant patients with cetuximab, higher late toxicity Subgroup: No significant sex-related differences	Weidhaas et al. (2026)
PATHOS (NCT02215265)	III	Transoral surgery + neck dissection, postoperative risk-stratified de-intensified adjuvant treatment	iENE has low sensitivity (up to 56.4%) and high specificity (at worst 70.9%) for predicting pENE; sensitivity/specificity improved after excluding suboptimal imaging and recent biopsy; inter-rater agreement significantly enhanced after training	HPV-positive as the core subgroup; 80.4% male, 19.6% female, no sex differences reported	Evans et al. (2026)
CTRI/2024/04/064,991	Not applicable	Not applicable (Observational study)	12% HPV positivity (HPV 58: n = 2; HPV 59: n = 1); No HPV 16/18 detected; Significant association with high alcohol consumption and tobacco chewing	Biomarkers: HPV 58/59; Subgroup: All HPV-positive patients were male OSCC cases (mean age 50.7 years); Tumor sites: oral cavity (tongue, buccal, labial mucosa); 66.7% with smokeless tobacco use	Maity et al. (2025)
Retrospective analysis (no specific trial name)	Not applicable	Definitive RT ± chemotherapy (cisplatin or cetuximab); need for precise risk stratification to select low-risk patients for de-escalation (current de-escalation trials showed inferior results)	26-peptide signature predicts recurrence (C-index = 0.941); stratifies into low/intermediate/high risk (high-risk all recurred, low-risk no recurrence); outperforms clinicopathologic variables	26-peptide signature as prognostic biomarker; no significant sex difference (91.1% male, 8.9% female, P = 1)	Jackson et al. (2025)
Observational population-based study (non-interventional trial)	N/A	Not specified; recommends combined HPV and p16 testing for patient selection in de-escalation/escalation clinical trials	- AAIR of total OPSCC increased from 1.8 to 5.1/100,000 (2000–2020), driven by HPV+/p16+ subgroup (0.9–3.5/100,000) - HPV+/p16+ had highest 5-year OS (79.2%); HPV-/p16- lowest (35.1%) - HPV-/p16- AAIR decreased from 1.6 to 1.4/100,000 (2017–2020)	- HPV+/p16+ as favorable prognostic biomarker - Male:female ratio 2.6:1; males had higher HR in early UICC stage (1.56, p < 0.001) - HPV+/p16+ more common in tonsils (63.0%), non-smokers (32.5%), and no alcohol abuse (79.6%)	Lauritzen et al. (2024)
NCT04638465 (Nonrandomized controlled trial)	Not specified	Surgery ± adjuvant therapy; primary radiotherapy ± chemotherapy with reduced doses (50–60 Gy vs. 70 Gy References)	Higher radiotherapy dose associated with inferior QoL (lower FACT-HN) and greater depression burden (higher QIDS-SR); 5 deaths, 2 recurrences (median follow-up 2.2 years)	HPV + OPSCC; 98% male, 98% White; T4 tumors linked to worse outcomes; no sex-specific differences noted	Panwar et al. (2024)
Retrospective cohort study (COHOC, Denmark vs. Stiefel OPSCC Database, US)	N/A (observational study)	Regimen: Denmark - RT alone (30.3%), CRT (51.0%); US - CRT (49.8%), neoadjuvant chemo + RT/CRT (16.7%), surgery + RT/CRT (9.8%). De-escalation: Low-risk (T1-T2N0M0) - RT alone (Denmark 41.7% vs. US 19.6%), similar prognosis	Efficacy: 3-year OS - US 91% vs. Denmark 83% (unadjusted); no adjusted OS difference (HR 1.21, p = 0.23); better RFI in US (HR 1.74, p = 0.003); high-risk (III-IV) US better OS (HR 2.20, p = 0.004) and RFI (HR 2.80, p = 0.002). Resistance: Higher recurrence risk in Denmark (linked to more RT alone)	Biomarker: HPV+ (better prognosis, US 88.2% vs. Denmark 63.1%); HPV- (OS HR 2.56, p < 0.001). Subgroup: Low-risk (T1-T2N0M0) - similar prognosis; high-risk (III-IV) - US better outcome. Sex: More males in US (89.4% vs. 74.1%), no significant OS impact (HR 1.30, p = 0.06)	Carlander et al. (2024)
DAHANCA 19 (NCT00496652)	III	Primary (chemo-)radiotherapy with nimorazole; weekly cisplatin for stage III–IV; hyperfractionated RT (76 Gy/56 fractions) without cisplatin for T2-4N0. No de-escalation strategy evaluated	Efficacy: p16 IHC and HPV DNA equally prognostic for loco-regional failure and OS (HR∼0.22–0.25, p < 0.0001). Resistance: No outcome differences by HPV genotype (p = 0.9 for loco-regional failure, p = 0.7 for OS)	Biomarker: p16 IHC and HPV DNA highly concordant (98%); HPV16 most common (87%). Sex: Male predominance (82% overall); no significant sex-specific outcome differences reported	Lilja-Fischer et al. (2023)
NCT02675439, NCT03172936, NCT03010176	Not specified	STING agonist + anti-CTLA-4/anti-PD1 ICB; De-escalation considered for HPV-positive OPSCC	Efficacy: Tumor STING immunoexpression (THS) associated with better DFS in HPV16-positive patients (p = 0.047); Resistance: HPV16 E^7^ oncoprotein blocks cGAS-STING response	Biomarker: STING immunoexpression (THS); Subgroup: HPV16-positive patients benefit; Sex: Female patients have better OS (p = 0.001)	Biesaga et al. (2022)
Prospective observational cohort (no specific trial name)	Not applicable (observational study)	Treatment included RT, CRT, surgery, induction chemo + CRT; de-escalation not evaluated	95% concordance between pre-treatment cfDNA-HPV ddPCR and p16 IHC/tissue PCR; post-treatment detectable cfDNA-HPV at ∼13 weeks associated with lower 1-year TTP (50% vs. 93.2%, p = 0.001); persistently elevated or subsequent rise in cfDNA-HPV linked to recurrence	Biomarker: cfDNA-HPV copy number (predictive of recurrence/outcomes); Sex: 73 male, 31 female (no sex-specific outcomes reported)	Warlow et al. (2022)
NRG-HN002 (NCT02254278)	II	60 Gy IMRT + weekly cisplatin (6 weeks) vs. 60 Gy IMRT alone (5 weeks); Reduced RT dose (60 Gy) from standard 70 Gy	- IMRT + C: 2 years PFS 90.5% (met target), 1 year MDADI 85.30; IMRT: 2 years PFS 87.6% (did not meet target), 1 year MDADI 81.76 - Higher acute grade 3–4 AEs with IMRT + C (79.6% vs. 52.4%); similar late AEs (21.3% vs. 18.1%)	- Eligibility: p16-positive (HPV-associated), ≤10 pack-years smoking −84.0% male; no significant sex-based efficacy difference reported	Yom et al. (2021)
NRG Oncology RTOG 1016	III	Radiotherapy + cetuximab vs. radiotherapy + cisplatin; attempted cetuximab substitution for cisplatin to reduce toxicity	- Efficacy: Cetuximab inferior to cisplatin in OS (HR 1.45, 5-year OS 77.9% vs. 84.6%), PFS (HR 1.72, 5-year PFS 67.3% vs. 78.4%), and locoregional failure (HR 2.05, 5-year 17.3% vs. 9.9%) - Resistance: No significant difference in acute/late moderate-severe toxicity rates; cisplatin had higher acute toxicity burden (T-score 3.19 vs. 2.35)	- Biomarker: HPV-positive (p16 expression) - Subgroup: Cetuximab not non-inferior in all subgroups; worse outcomes in Zubrod PS 1 (HR 2.66) - Sex: Predominantly male (90%), female 10%; no significant sex-specific efficacy difference	Gillison et al. (2019)
PATHOS (Post-operative adjuvant treatment for HPV-positive tumours)	II/III	- Group A (low-risk): No adjuvant treatment - Group B (intermediate-risk): Randomised to standard RT (60Gy/30f) vs. reduced RT (50Gy/25f) - Group C (high-risk): Randomised to standard CRT (60Gy + cisplatin) vs. RT alone (60Gy)	Phase II primary: 12-month MDADI score (swallowing function); Phase III planned primary: Overall survival. Aim to maintain efficacy (OS, LC) while reducing toxicity (dysphagia)	Biomarker: HPV positivity (strong prognostic factor). Subgroup analysis by T stage, tumour subsite (tonsil, soft palate, tongue base); Sex not specified	Owadally et al. (2015)
ECOG-ACRIN E3311	II	Transoral surgery followed by risk-adjusted adjuvant therapy: Low-risk (T1-T2, N0-N1, negative/close margins, no ENE) observed (Arm A); Intermediate-risk (T1-T2, negative margins, N1-N2 with ≤1 mm ENE or ≤4 nodes) randomized to 50 Gy (Arm B) vs. 60 Gy (Arm C); High-risk (positive margins, >1 mm ENE, ≥5 nodes) received RT + weekly cisplatin (Arm D)	Low positive margins (3.8%), grade III/IV oropharyngeal bleeding (5.9%), grade V bleeding (0.2%); composite QA endpoint 8.9%, supporting safety of transoral surgery in HPV + OPC	HPV + OPC as target; Male (89.9%) vs. Female (10.1%) with no significant difference in outcomes; TORS vs. TLM showed similar margin/bleeding rates (p = 0.89/p = 0.24); Surgeon case volume (<5 vs. ≥ 5 patients) not associated with significant differences in margins/bleeding (p = 0.56/p = 0.42)	Ferris et al. (2020)

## 7 Contributions and limitations of current research models

### 7.1 Patient-derived organoids and xenografts

Patient-derived organoids (PDOs) recapitulate the genetic features of parental head and neck tumors; targeted sequencing demonstrated that three out of four paired HNCOs showed >50% mutational concordance with their tumors of origin, although clonal evolution during long-term passaging may influence therapeutic responses (Choi et al., 2023). They can distinguish inter-patient sensitivities to cisplatin, carboplatin, cetuximab, and radiotherapy, with *in vitro* drug responses mirroring clinical treatment outcomes (Driehuis et al., 2019). In 3D invasion assays, HNSCC PDOs upregulate a YAP-centered transcriptional program under invasive conditions, with collective invasion associated with elevated YAP target expression and nuclear translocation (Haughton et al., 2024), while parallel chemoresistance-focused studies have identified YAP/TAZ pathway activation as a resistance-associated transcriptional signature (Wang et al., 2026). PDOs are particularly suited for longitudinal monitoring of clonal evolution under therapeutic pressure: head and neck cancer organoids (HNCOs) display clonal dynamics with serial passaging, where late-passage organoids often show altered sensitivity to radiation and chemotherapy, enabling functional validation of clonal evolution on treatment response in *ex vivo* models (Choi et al., 2023). This capability is exemplified by the tracking of CDCP1-high subpopulations, which were identified through transcriptomic profiling of OSCC PDOs as key drivers of cisplatin resistance via Wnt/β-catenin-mediated stemness, and whose therapeutic targeting with pH-sensitive nanoparticles restored chemosensitivity in patient-derived xenograft (PDX) models (Wang et al., 2026). PDXs initially retain human stromal components of the original tumor, including cancer-associated fibroblasts and vasculature; however, the human stroma is progressively replaced by murine stroma during serial passaging (Qian et al., 2022). PDXs also preserve key genetic features of HPV + HNSCC—including PIK3CA mutations, TRAF3 deletion, absence of EGFR amplifications, and E6/E7 viral oncogene expression—but require immunodeficient host mice (e.g., NSG), meaning they lack a functional adaptive immune system and thus preclude the study of T cell-dependent immune surveillance and immunotherapy response (Facompre et al., 2020).

### 7.2 Immunocompetent mouse models: The MOC1-HPV paradigm

The MOC1-HPV model, generated by retroviral transduction of HPV16 E^6^/E7 oncogenes into the murine oral carcinoma (MOC1) cell line, provides a syngeneic, immunocompetent C57Bl/6NCrl-based system that recapitulates key features of HPV-positive oral squamous cell carcinoma, including stable viral oncogene expression, elevated radiosensitivity, and an active tumor immune microenvironment (Modic et al., 2023). Studies in the parental MOC1 model have shown that the WEE1 inhibitor AZD1775 not only radiosensitizes tumor cells through abrogation of the G2/M checkpoint and induction of mitotic catastrophe, but also enhances granzyme B-dependent CD8^+^ T lymphocyte killing and synergizes with PD-1 immune checkpoint blockade to improve tumor control and complete rejection rates—findings impossible in immunodeficient systems (Patel et al., 2019). WEE1 inhibition also sensitizes murine and human HNSCC cells to NK cell-mediated lysis, providing complementary immune-based mechanisms of tumor clearance (Friedman et al., 2018). More recent investigations in the MOC1-HPV K1 model have revealed heterogeneity in anti-tumor immune responses: responding tumors are enriched with effector memory T cells, whereas non-responders exhibit increased infiltration of myeloid-derived suppressor cells (MDSCs) and monocyte-derived dendritic cells (Pisljar et al., 2025). However, differences from human disease—including retroviral rather than natural HPV integration, lack of viral genome fragmentation, and a murine oral rather than human oropharyngeal microenvironment—mean that MOC1-HPV is best positioned for mechanistic validation rather than direct clinical prediction, particularly given the intra-model variability in treatment responses that mirrors the clinical heterogeneity observed in HPV-positive patients (Modic et al., 2023).

### 7.3 High-throughput sequencing and bioinformatics

Whole-exome and transcriptome sequencing have systematically catalogued the mutational landscape of HPV-positive OPSCC, revealing distinct genomic alterations linked to treatment resistance. These include upregulated protein levels of ATR pathway effectors, in the absence of increased ATM effector signaling, suggesting selective dependency on the ATR-CHK1 axis (Kono et al., 2020). Additionally, high intratumoral genomic heterogeneity and copy number alterations are strongly associated with poor recurrence-free survival, and identified loss of distal 11q, which encodes key double-strand break repair genes including ATM and MRE11A(Patel et al., 2021). Machine-learning-based integrative analyses are beginning to refine patient stratification. For example, combining clinically established p16/HPV DNA status with a 24-miRNA-defined molecular phenotype can improve clinically relevant risk stratification of HNSCC patients, which may guide future treatment de-escalation strategies (Hess et al., 2022).

## 8 Key challenges, controversies, and translational barriers

### 8.1 Lack of a unified molecular taxonomy of resistance

Numerous resistance-associated features—MACROD2 deletion and circ_0002722/YAP/TAZ pathway activation—have been described in isolation, yet a unified molecular taxonomy of resistance remains elusive. While histological and genetic criteria have recently been used to define clinically relevant high-risk subgroups of HPV-positive OPSCC(Suchan et al., 2025). Nevertheless, the foundation for such a classification is rapidly being built through integrative multi-omics efforts: transcriptomic and proteomic profiling has identified four distinct molecular clusters of HPV-associated OPSCC corresponding to different mechanisms of chemoresistance, validated in an external cohort (Lu, 2025). The KRAS-variant’s context-dependent associations—conferring benefit when cetuximab is added to cisplatin-based chemoradiotherapy (RTOG 0522) but only identifying a subgroup for whom cetuximab is not inferior to cisplatin when substituted for the latter (RTOG 1016) — exemplify the challenge of applying single biomarkers across distinct therapeutic scenarios (Weidhaas et al., 2026). These observations underscore the imperative for prospective multi-omics biomarker integration, wherein liquid biopsy-based approaches that provide independent prognostic value beyond p16 and HPV status may serve as ideal platforms for future validation.

### 8.2 Translational gap from discovery to clinic

MACROD2 loss drives radioresistance but does not affect cisplatin sensitivity in HPV-positive HNSCC cell lines—an effect accompanied by elevated hypoxia levels and an altered DNA damage response—illustrating treatment-specific biomarker behavior (Dawson et al., 2024). WEE1 inhibition with AZD1775 not only radiosensitizes tumor cells through abrogation of the G2/M checkpoint and induction of mitotic catastrophe, but also enhances CD8^+^ T cell-dependent tumor cell killing and synergizes with PD-1 immune checkpoint blockade—findings demonstrated in the MOC1 syngeneic mouse model that highlight the immunomodulatory potential of this approach (Patel et al., 2019). The combination of WEE1 inhibition and radiation also potentiates CD8^+^ T cell-dependent antitumour activity via dendritic cell activation (Wang et al., 2020). However, these immunomodulatory effects require further validation in the MOC1-HPV K1 model, where significant heterogeneity in antitumor immune responses has been observed—responder tumors are enriched with effector memory T cells, while non-responders exhibit increased infiltration of neutrophils (MDSCs) and monocyte-derived dendritic cells (Pisljar et al., 2025). While PDOs, PDXs, and syngeneic models such as MOC1-HPV have advanced substantially, each retains inherent limitations that constrain clinical predictive value. PDOs lack functional vasculature and the complete immune repertoire, limiting their capacity to fully model the tumor-immune-stroma crosstalk (Haughton et al., 2024). PDXs retain HPV oncogene expression and key genetic features of the original tumor, but are subject to murine stromal replacement and depend on immunodeficient hosts, precluding the study of adaptive immune surveillance (Facompre et al., 2020). Syngeneic GEMMs such as MOC1-HPV provide an intact murine immune system that enables mechanistic validation of immunomodulatory strategies, yet their subcutaneous localization, murine viral integration patterns, and immunocompetent background limit their capacity to directly predict clinical outcomes (Modic et al., 2023).

### 8.3 Protracted biomarker validation

Most candidate biomarkers—DDR genes, non-coding RNAs, CSC markers, ctDNA variants—derive from retrospective analyses or small case–control studies. MACROD2’s association with radioresistance, for instance, is based on limited-sample gene expression analysis and awaits prospective validation (Campo et al., 2024). Liquid biopsy technologies offer non-invasive tools for dynamic monitoring, but standardization and clinical validation are contingent on prospective cohort development (Damodaran et al., 2025; Dranova, 2026).

### 8.4 Balancing resistance circumvention with toxicity

De-escalation strategies aim to mitigate late toxicity, but in RTOG 1016, substituting cetuximab for cisplatin resulted in inferior survival, partly due to genetic factors (Yacoub et al., 2025). Critically, these trials were not designed to prospectively collect biomarkers that could identify patients suitable for reduced-intensity treatment without excess resistance risk. Optimizing therapeutic windows through scheduling, dosing, or nanoparticle delivery systems is a key translational focus (Yacoub et al., 2025).

## 9 Future directions and translational prospects

### 9.1 Precision strategies based on the resistance atlas

ATR inhibitors can enhance radiosensitivity and, in preclinical models, promote antitumor immunity through cGAS–STING/interferon signaling and modulation of the tumor microenvironment. WEE1 inhibitors also enhance radiosensitivity, and emerging evidence suggests that they may augment antitumor immunity in a context-dependent manner (Lee et al., 2025). Nanoparticle-based delivery systems may offer a strategy to improve drug delivery and potentially overcome resistance, but their relevance to HPV-positive tumors and E6/E7-driven DDR aberrations remains to be specifically tested in HPV-relevant models (Chenxi et al., 2025).

### 9.2 Novel biomarkers for resistance surveillance

Liquid biopsy modalities—ctDNA, circulating tumor cells, and exosomes—are emerging as repeatable, non-invasive tools for longitudinal monitoring of resistance dynamics (Pandey and Yadav, 2025). Plasma HPV DNA persistence or reappearance has been associated with treatment failure, though sensitivity and specificity require uniform validation in large prospective cohorts (Wotman et al., 2025). Exosomal non-coding RNAs may be suited for longitudinal tracking but await rigorous prospective confirmation (Li et al., 2021). DDR proteins (ATM, ATR, γH2AX) and epigenetic marks are candidate surveillance biomarkers (Williams and Zhang, 2021).

### 9.3 Prospective clinical trials for resistance-directed regimens

The lessons from trials such as RTOG 1016 suggest that treatment de-escalation in HPV-positive OPSCC requires careful patient selection, and that improved predictive and prognostic biomarkers should be incorporated into future trial design and stratification (Rosenberg and Vokes, 2021). Future trials should adopt adaptive designs that permit dynamic treatment modification—for instance, adding an ATR inhibitor or immune checkpoint blockade upon detection of radioresistance signals. Basket or umbrella trial designs can enroll patients with different resistance mechanisms under a unified framework, accelerating regimen identification (Kaizer et al., 2023).

## 10 Conclusion

Therapeutic resistance in HPV-positive OPSCC is a multidimensional, dynamically evolving process orchestrated by DDR reprogramming, epigenetic plasticity, CSC maintenance, and an immunosuppressive, hypoxic microenvironment (Tsai et al., 2024; Lu, 2025). Cross-regulation among these mechanisms is evident: circ_0002722-driven YAP/TAZ activation confers platinum resistance in oral squamous cell carcinoma through the miR-1305/YAP regulatory axis, with verteporfin-mediated YAP inhibition synergizing with cisplatin to overcome. Parallel evidence demonstrates that YAP/TAZ pathway activation is associated with both cisplatin resistance and CSC stemness maintenance in head and neck cancer, as identified through patient-derived organoid-based transcriptomic profiling (Yu et al., 2025). The field urgently requires a prospectively validated, integrative molecular taxonomy of resistance to enable cross-study comparison and translation. Recent progress supports the feasibility of such a taxonomy: integrative multi-omics profiling of HPV-associated OPSCC has identified four distinct molecular clusters with divergent transcriptional regulatory networks, tumor immune microenvironments, and clinical outcomes, plausibly corresponding to different mechanisms of chemoresistance (Lu, 2025). Priorities include: establishing prospective-cohort-based biomarker validation systems—as exemplified by the demonstration that TTMV HPV DNA-guided surveillance detects 40% of recurrences earlier than standard-of-care methods with a median lead time of 132 days, albeit with sensitivity limited to HPV16 and detectable pretreatment TTMV, underscoring the need for multi-analyte biomarker panels (Rettig et al., 2025); co-targeting the DDR and immune escape—a strategy supported by the mechanistic interplay between DDR defects and immune signaling via cGAS-STING activation and immune checkpoint modulation (Marcut et al., 2025); and employing advanced nanoparticle delivery systems—including CD44-targeted virus-mimicking nanomedicines that achieve 12-fold enhanced anti-CSC efficacy when combined with cisplatin (Chen et al., 2025) and acidic-responsive nanoformulations of WEE1 inhibitors that sensitize ferroptosis to enhance immunotherapy (Zhou et al., 2025)—to achieve effective drug exposure within resistant tumors while minimizing systemic toxicity. Addressing these challenges will be essential to realize the promise of precision oncology for patients with HPV-positive OPSCC.
